# Supplementary material for: A Highly Sensitive, Low Creep Hydrogel Sensor for Plant Growth Monitoring
Source: Sensors (Basel). 2024 Sep 25;24(19):6197. doi: 10.3390/s24196197 (PMC11478791; doi:10.3390/s24196197)
Supplement: Supplementary file 1 [file sensors-24-06197-s001.zip › sensors-3151182-supplementary.pdf]

Supplementary Materials for

A Highly Sensitive, Low Creep Hydrogel Sensor for Plant Growth Monitoring

Haoyan Xu †, Guangyao Zhang †, Wensheng Wang, Chenrui Sun, Hanyu Wang, Han Wu * and Zhuangzhi Sun *

Province Key Laboratory of Forestry Intelligent Equipment Engineering, College of Mechanical and Electrical Engineering, Northeast Forestry University, Harbin 150000, China

* Correspondence: whan@nefu.edu.cn (H.W.); sunzhuangzhi@nefu.edu.cn (Z.S.)

† These authors contributed equally to this work.

Brief description of what this file includes:

Figure S1. Mechanical properties of SNaPVA-Sensor.

Note S1. SNaPVA-Sensor monitors plant growth mechanisms.

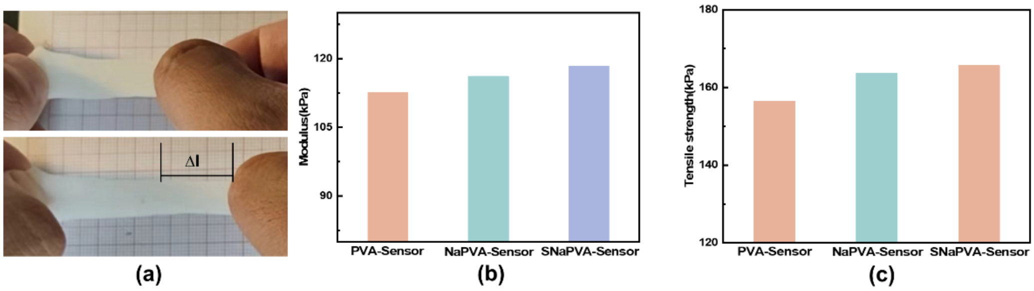

Figure S1. Mechanical properties of SNaPVA-Sensor. (a) Tensile condition of SNaPVA-Sensor; (b) Young's modulus of SNaPVA-Sensor; (c) Tensile strength of SNaPVA-Sensor.

Note S1. SNaPVA-Sensor monitors plant growth mechanisms.

Using the strain-sensitive properties of SNaPVA-Sensor, it can be attached or wrapped on the surface of plant stems, trunks or other parts. The specific sensing mechanism is as follows: When the plant grows, the diameter or width will increase, and the surface area of the stem or trunk will increase, causing the hydrogel sensor to be stretched. During the stretching process, the internal network structure of the hydrogel is squeezed, the network density increases, and the ion movement rate decreases, resulting in increased resistance. By monitoring the deformation of SNaPVA-Sensor, the dynamic process of plant growth can be tracked.
